# An Alternative Microbiological Validation for an Online Water Bioburden Analyzer

**DOI:** 10.1093/jaoacint/qsae050

**Published:** 2024-06-25

**Authors:** Olivia L Venhuizen, Cynthia E Martindale, Feng Jin Liew, James Cannon, Arundhati Samanta, Mike J Scaramozzino

**Affiliations:** Process Development, Cellular Sciences, Amgen, One Amgen Center Drive B15 MS 1A, Thousand Oaks, CA 91320, United States; Applied Rapid Microbiology Specialists, Ltd, 1634 Sunset St, Longmont, CO 80501, United States; Marketing, Mettler-Toledo, 900 Middlesex Turnpike, BLD 8, Billerica, MA 01821, United States; Marketing, Mettler-Toledo, 900 Middlesex Turnpike, BLD 8, Billerica, MA 01821, United States; Marketing, Mettler-Toledo, 900 Middlesex Turnpike, BLD 8, Billerica, MA 01821, United States; Marketing, Mettler-Toledo, 900 Middlesex Turnpike, BLD 8, Billerica, MA 01821, United States

## Abstract

**Background:**

The Mettler-Toledo 7000RMS analyzer is a bio-fluorescent particle counter (BFPC) used to monitor real-time bioburden results from purified water (PW).

**Objective:**

Validation of the analyzer using 13 microorganisms and a low-intensity, fluorescent, polystyrene bead.

**Methods:**

During the execution of the validation, a laboratory water system that met PW quality standards was connected to the 7000RMS, and a syringe pump was used to introduce various concentrations of microorganisms and fluorescent polystyrene beads to the analyzer. Samples were collected and tested via the traditional membrane filtration (MF) method and the colony-forming unit (CFU) plate count results were compared to the auto-fluorescent unit (AFU) of the 7000RMS analyzer. The validation study was designed to follow the guidance in United States Pharmacopeia (USP) Chapter <1223>, European Pharmacopeia (EP) Chapter 5.1.6, and parenteral drug association (PDA) Technical Report 33. Concepts and strategies were adapted from EP Chapter 2.6.12 Microbiological Examination of Non-Sterile Products: Microbial Enumeration Tests, EP Chapter 10.2, EP Chapter 2.6.1 Sterility, USP Chapter <61> Microbiological Examination of Non-Sterile Products: Microbial Enumeration Tests, USP Chapter <71> Sterility Tests, and Japanese Pharmacopoeia (JP) General Information Chapter G8 Water: Quality Control of Water for Pharmaceutical Use.

**Results:**

All pre-determined validation acceptance criteria for accuracy, specificity, precision, LOD, LOQ, linearity, and range were met.

**Conclusions:**

The 7000RMS demonstrated performance equivalence to the MF method per USP <1223> but characteristically lacked correlation to the CFU.

**Highlights:**

This validation approach highlights the superior capabilities of the 7000RMS when compared against the traditional compendial MF testing method for PW.

The 7000RMS is intended as an alternative method to the current membrane filtration (MF) method for analyzing microbial content in PW and water for injection (WFI) samples. The 7000RMS technology has advantages when compared to the MF method. These advantages are summarized in Table 1.

A validation of the 7000RMS was conducted to determine if the instrument is a suitable alternative to the MF method for monitoring PW. This validation preceded any comparative testing conducted on the water loops themselves (e.g., PQ testing and/or in situ decision equivalence testing).

The numerous limitations of traditional microbiological test methods are the main drivers for innovation in microbiological testing. The increasing need for faster results eventually led to the development of rapid microbiological methods (RMMs) for direct analysis, which use the entire microbial cell for detection; these technologies include laser-induced fluorescence (LIF), which exploits the intrinsic fluorescent properties of metabolites produced by the cell. No previous validation studies using Mie scattering and LIF have been done. Therefore, this validation approach can set the stage for end-users to adopt their own validation strategies for this online water bioburden analyzer (OWBA) technology.

The Mettler-Toledo Thornton 7000RMS uses LIF to detect and enumerate microorganisms in the presence of water. All microorganisms use metabolites such as nicotinamide adenine dinucleotide (NADH) and riboflavin to regulate their growth and development. When exposed to the 7000RMS 405 nm laser, these metabolites are excited to produce intrinsic fluorescence emissions. As the sample flows through the optical pathway, one detector measures the fluorescence emissions while another detects and sizes particles via the Mie light-scattering principle. Using a proprietary set of algorithms, data from each detector is processed to report the results. Fluorescent particles of a certain size range are determined by the 7000RMS to be microorganisms and are reported as an auto-fluorescent unit (AFU). Particles that do not fluoresce are reported as inert particles.

The 7000RMS system provides real-time microbial content results for water samples. When operating in online mode, this system provides continuous and rapid results at a rate of 1 mL every 2.2 s (30 mL/min) without needing reagents or consumables. The 7000RMS may also be operated in sample mode, allowing for the analysis of water collected in a sample bottle. The current MF method requires collection of a discrete sample, transport to a laboratory, and use of various consumables to complete the analysis. The time to result for MF is lengthy requiring an incubation of 5–7 days from the date of plating.

The most common guidance documents discussing the validation of alternative microbiological methods in the pharmaceutical industry are United States Pharmacopeia (USP) chapter <1223> (1), European Pharmacopeia (EP) chapter 5.1.6 (2), and the Parenteral Drug Association (PDA) Technical Report (TR) No.33 (3). Additional pharmaceutical industry guidance documents were used in this validation (4–8, 10–13). The validation covered all validation parameters in these guidance documents to demonstrate that the 7000RMS bio-fluorescence particle counter (BFPC) was suitable to use in place of (or in augmentation to) the traditional growth-based membrane filtration (MF) method for purified water (PW).

Following these guidance documents can prove to be challenging, however, as many of the validation parameters use a comparison of results between the alternative method and the method already in place. In this case, a comparison would be made of count results from the 7000RMS and the MF method. This is much like comparing apples and oranges. The AFU count reported by the 7000RMS is not equivalent to a colony-forming unit (CFU) count from the MF method and, therefore, a direct comparison of counts is impractical.

The presence of an AFU requires only a particle with the proper fluorescent signatures for the biomarkers of interest (e.g., NADH and riboflavin). However, the presence of a CFU on the MF method requires that the incubation conditions and nutritional requirements of the media are met sufficiently to provide for growth of cells to a large enough size for detection. These fundamental differences in detection result in a non-equivalence between the AFU and the CFU (8, 9). This non-equivalence means that a traditional comparison (between CFU vs. AFU) is not possible when applying validation parameters for accuracy, LOD, LOQ, linearity and range. Therefore, while microorganism data was collected and reported following strategies in the guidance documents, beads were used as an absolute determination of the validation parameter.

The validation utilized 13 microorganisms and one low-intensity fluorescent bead. The rationale for the selection of each and where it was used within the 7000RMS validation are listed in Table 2.

**Table 1.** 7000RMS advantages and MF limitations

Type	7000RMS capability	MF capability	7000RMS advantage
Time to result (1 mL)	∼2 seconds	5–7 days	Faster time to result
Type of analysis	Continuous	Discrete	More results provide for an increased chance to detect microorganisms
Sample handling	No sampling (online)	Required	Decreases the number of false-positive events due to sample handling
Availability of result	Immediate (SCADA connectivity)^a^	Delayed (analyst-driven)	Increased visibility to real-time results by operations staff
Reaction time to limit violation condition	Immediate (SCADA connectivity)	Delayed (5–7 days for result)	Real-time decision-making and corrections are possible
Trending	Immediate and continuous	Delayed (monthly/quarterly)	View and react to trends in real time
Consumable usage	None required	Required	Reduced cost and improved convenience

a SCADA = Supervisory Control and Data Acquisition.

**Table 2.** Microorganisms and bead used in 7000RMS validation

Microorganism (source)	Rationale	Specificity	Accuracy	Precision	LOD	LOQ	Linearity	Range	Equivalency	Correlation
*Pseudomonas. paraeruginosa* (ATCC 9027)	Representative non-fermenting Gram-negative rod which can be present in water	x	x	x	x	x	x	x	x	x
	European Pharmacopoeia (4, 13)									
	United States Pharmacopoeia (5, 6)									
	Japanese Pharmacopoeia (7)									
*Bacillus spizizenii* (ATCC 6633)	Representative Gram-positive rod	x	x		x		x		x	
	European Pharmacopoeia (4, 13)									
	United States Pharmacopoeia (5, 6)									
*Bacillus spizizenii*, heat-shocked (ATCC 6633)	Representative spore former	x	x	x	x	x	x	x	x	x
	European Pharmacopoeia (4, 13)									
	United States Pharmacopoeia (5, 6)									
*Ralstonia pickettii* (in-house isolate No. 1)	Common microorganism found in water. In-house source No. 1	x		x	x	x	x	x	x	x
*Ralstonia pickettii* (in-house isolate No. 2)	Common microorganism found in water. In-house source No. 2	x	x							x
*Staphylococcus aureus* (ATCC 6538)	Representative Gram-positive cocci	x	x	x	x	x	x	x	x	x
	European Pharmacopoeia (4, 13)									
	United States Pharmacopoeia (5, 6)									
	Japanese Pharmacopoeia (7)									
*Stenotrophomonas maltophilia* (ATCC 13637)	Common microorganism found in water	x	x	x	x	x	x	x	x	x
*Pseudomonas fluorescens—*starved (NBRC 15911)	Japanese Pharmacopoeia (7)	x	x	x	x	x	x	x	x	x
*Pseudomonas gluocanolyticus* (in-house isolate)	Common microorganism found in water. In-house source.	x	x		x		x		x	
*Escherichia coli* (ATCC 8937)	Representative coliform, which can be present in water	x	x	x	x	x	x	x	x	x
*Sphingomonas paucimobiilis* (in-house isolate)	Common microorganism found in water. In-house source.	x	x	x	x	x	x	x	x	x
	Organisms involved in FDA recalls 1995–2002									
*Burkholderia cepacia* (ATCC 25416)	Common microorganism found in water.	x	x	x	x	x	x	x	x	x
	Organisms involved in FDA recalls 1995–2002									
Mixed culture (equal portions)*: Pseudomonas paraeruginosa* (ATCC 9027) and *Staphylococcus aureus* (ATCC 6538)	PDA Technical Report 33 (3)	x			x				x	
Yellow, low-intensity, 0.7–0.9 µm, fluorescent beads (Spherotech Cat. No. FL-0852–2)	Suitable fluorescence and size, which are constant in water over analysis period	x	x	x	x	x	x	x	x	

## Experimental

Microbial method validations typically consist of spiking microorganisms into samples and then testing them by the current and alternative methods. However, bringing microorganisms into a pharmaceutical facility and spiking them into a water system is in violation of good manufacturing practices (GMPs). Therefore, a Barnstead GenPure^TM^ Ultra-Pure water generation system served as the water source during laboratory validation testing. Water produced by this system met testing standards for PW and was 0.05 µm (50 nm) filtered. The term “water” is used throughout this paper to describe this.

Testing was conducted using both 7000RMS operating modes, online mode and sample mode. These are described further in the testing section. The apparatus and materials for each testing mode are listed in Tables 3 and 4.

**Table 3.** Apparatus

Apparatus	Online mode	Sample mode
Real-time monitoring system, Mettler-Toledo 7000RMS	x	x
Barnstead GenPure Pro, Cat. No. 41862057, modified to allow for both on-demand water as well as continuous water feed. A Fiberflo^®^ 0.05 µm filter (listed below) was added to the on-demand dispenser.	x	x
Harvard Apparatus, Ultra PHD Infuse Only	x	
Hamilton 100 mL gastight syringe, model 1100 TLL, PTFE Luer lock	x	
Fiberflo 0.05 µm pore size hollow fiber capsule filter	x	
Sample input tube (PVC Tygon) with connectors 1/16” ID x 1/8” OD, Mettler-Toledo		x
Sample syringe needle with Luer lock (304 SS), Mettler-Toledo		x

**Table 4.** Reagents and materials

Reagents and materials	Online mode	Sample mode
Veltek Associates, Inc., Steri-Perox^®^ 6% hydrogen peroxide (H_2_O_2_; Malvern, PA, United States)	x	x
BD DIFCO™ Reasoner’s 2 agar (R2A; Franklin Lakes, NJ, United States)	x	x
BD™ prepared sterile pack settling plated tryptic soy agar (Franklin Lakes, NJ, United States)	x *(microorganisms only)*	
BD™ tryptic soy broth (TSB), 10 mL (Franklin Lakes, NJ, United States)	x *(microorganisms only)*	
PALL^®^ 0.45 µm cellulose ester filter membrane micro-funnel (Port Washington, NY, United States)	x	x
Challenge microorganisms and beads as listed in Table 1	x	x

Aseptic technique and particle control should not be confused. Sterile materials can still contain a high number of particles. Since the 7000RMS defines AFU counts by examining whether a particle is of the appropriate size and fluorescence spectrum, there is always the possibility that the laboratory environment or testing process adds particles which could skew the results in favor of the 7000RMS. Particulates are ubiquitous in the environment and are extremely difficult to eliminate in a laboratory setting. Therefore, to ensure that the counts from the 7000RMS were related to the microorganism or bead, rather than an artifact from the laboratory conditions, the following particulate control measures were implemented during the validation:

1. Only materials constructed of sterilized glass or polyethylene terephthalate glycol (PETG) were used for testing.
2. All containers and syringes were filled with water and soaked overnight.
3. On the day of use, items were triple rinsed with water and tested by the 7000RMS to ensure that they provided for acceptably low AFU counts (generally 0–1 AFU/10 mL).
4. Dilution tubes and pipette tips were thoroughly rinsed with water prior to use.
5. The use of disinfectants was curtailed for the setup and preparation steps.

### Sample Preparation

**Figure 1.**
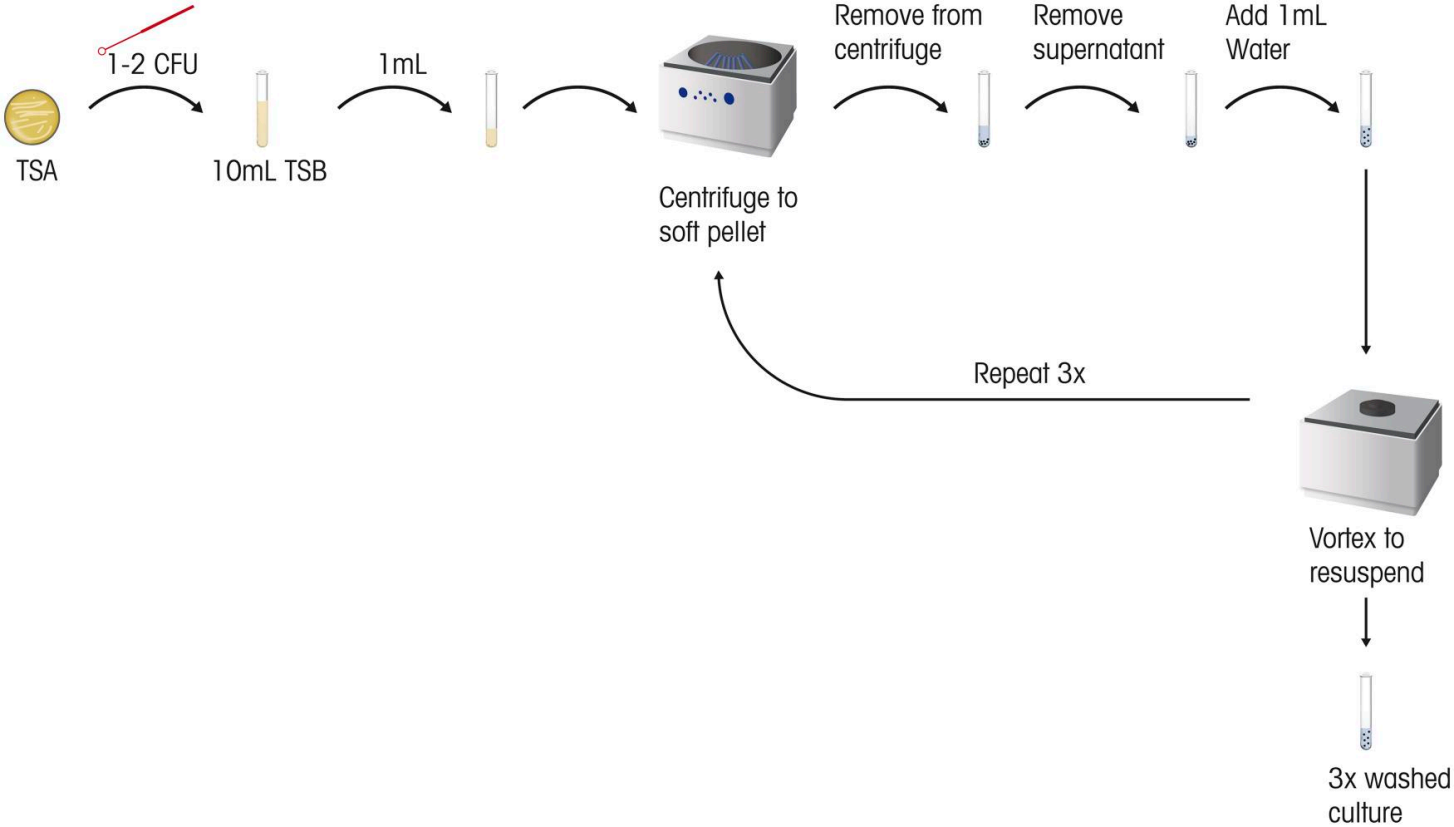
Microorganism growth and preparation.

- *Microorganisms.—*Microorganism growth was started approximately 2–3 days in advance of testing. The expected titers of each microorganism were characterized by growth studies executed before the validation. The microorganism growth steps are found in Figure 1. The growth conditions and durations established for each microorganism remained consistent throughout the validation.
     Previously frozen or freeze-dried culture isolates of the challenge microorganism of interest were streaked for isolation onto TSA plates and then incubated at 30–35°C for 24–48 h. Growth on the agar plate was examined for purity before transferring one or more colonies, using a 1 µL loop, to sterile TSB that was incubated at 30–35°C for 18–24 h. At the end of the incubation period, the broth culture was transferred to a 1.5 mL microcentrifuge tube, pelleted down, and resuspended in water. The culture was washed with water a total of three times. The 3×-washed microorganism was diluted with water to a calculated concentration of 6.0 × 10^3^ CFU/mL to create the microorganism inoculum.
     The microorganism inoculum concentration was determined from samples collected from the syringe at Time zero (T_0_) and at the end (T_end_) of the testing session. These aliquots are shown in Figure 4, as Step 8 and Step 12, respectively. Samples were diluted 1:10 and 1:100 using water. For *B. spizizenii* and *P. glucanolyticus*, where low CFU counts were expected, the aliquot was also tested neat. 1 mL of each dilution was transferred to a sterile filter funnel containing a 0.45 µm gridded cellulose-ester membrane filter. Vacuum pressure was applied to draw the sample through the membrane filter, which was then aseptically placed onto Reasoner’s 2 agar (R2A). The R2A plate was allowed to incubate for 5–7 days at 30–35°C.
- *Beads.—*The beads from the vendor were received in a vial at high titers of 10^10^ beads/mL. To ensure sufficient homogeneity, beads were both sonicated and mixed on a vortex mixer several times prior to creating a working stock solution of 10^7^ beads/mL. To ensure dilutions remained as accurate as possible to the vendor concentrations, the principle of 1 mg water being equivalent to 1 mL was used to determine the beads per unit volume (mL). Measurements were performed using calibrated balances and micropipettors. The working stock solution was subsequently diluted with PW to the required test concentrations to create the bead inoculum (refer to Table 5). Where the bead inoculum was to be used for online testing, a 200 mL preparation was created and 93 mL transferred to a syringe. Where the bead inoculum was used for sample mode testing, a volume of 5 mL was created in a triple-rinsed glass dilution tube.

**Table 5.** Inoculum concentrations

Testing mode	Challenge type	Inoculum concentration
Online	Beads at concentrations 50–10 000 per 10 mL	6.0 × 10^3^ beads/mL
Online	Beads at concentrations 5–1000 per 10 mL	6.0 × 10^2^ beads/mL
Online	Beads at concentrations 5–50 per 100 mL	3.0 × 10^1^ beads/mL
Online	Beads at concentrations 5–1000 per 100 mL	3.0 × 10^2^ beads/mL
Online	Beads at concentrations 250–10 000 per 100 mL	3.0 × 10^3^ beads/mL
Sample	1 mL sample volume tests	3.0 × 10^5^ and 3.0 × 10^3^ beads/mL
Sample	10 mL sample volume tests	3.0 × 10^5^, 3.0 x 10^3^ and 3.0 × 10^2^ beads/mL

### Analysis

Two different modes of operation (online and sample) are possible with the 7000RMS. The online mode is the operating mode used for water loop monitoring. This mode allows for continuous analysis of water. The sample mode is the 7000RMS operating mode used for discrete sample testing (e.g., those collected in a sample container). This mode allows for the analysis of water from a collection bottle. Both operational modes were utilized for validation testing as shown in Table 6.

**Table 6.** Summary of operational modes by validation parameters

Parameter	Microorganisms	Beads	
	Online mode	Online mode	Sample mode
Accuracy	x	x	x
Specificity	x		
Precision	x	x	x
LOD	x	x	x
LOQ	x	x	x
Linearity	x	x	x
Range	x	x	x
Correlation	x	x

**Figure 2.**
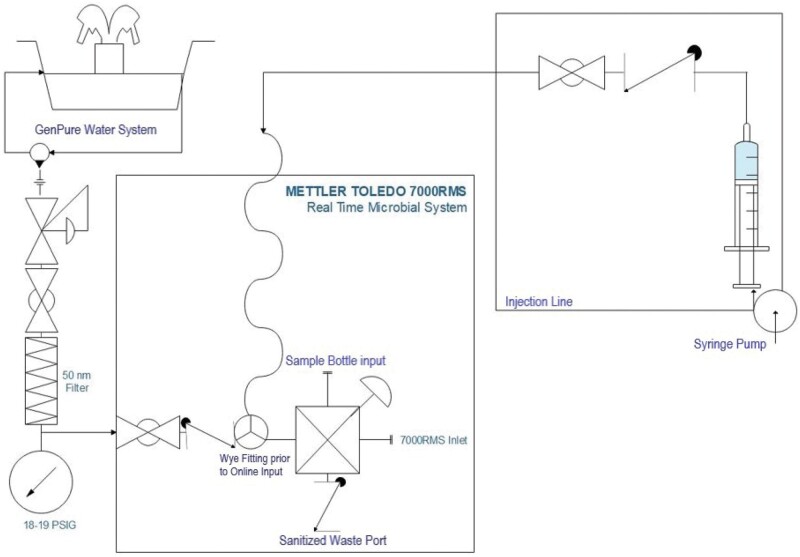
Online 7000RMS testing configuration.

- *Online mode.—*Online mode testing was performed to mimic the instrument's operation when installed on a pharmaceutical water system. In these cases, the 7000RMS system is installed in the water loop and a portion of the water is diverted into the instrument for analysis. Following analysis, the tested water is sent to a drain line as waste. The validation in this article was conducted in a laboratory due to the use of live microorganisms, which cannot be brought into a pharmaceutical manufacturing environment. As such, this validation did not cover any performance qualification activities, which would demonstrate the suitability of the actual 7000RMS installation in the pharmaceutical water system itself (e.g., in situ parallel or interferent testing).
     For testing conducted using the online mode, the 7000RMS was connected to a GenPure water system in the configuration depicted in Figure 2.
     Water pressure from the GenPure system was regulated to 18–19 PSIG, passed through a 50 nm (0.05 µm) hollow fiber filter, and ran to a wye-fitting. One side of this fitting was connected to the online input on the 7000RMS four-way valve, while the other side of the wye-fitting was connected to the injection line. The injection line, containing an on-off valve, was connected to a syringe seated in a syringe pump infuser.
     Both microorganisms and beads were tested using 7000RMS online mode during this validation. The specific analysis for each type of test are further described in sections (b) *Microorganism testing* and (c) *Bead testing*.
- *Microorganism testing.—*The syringe contained a prepared inoculum containing the microorganism to be tested. The syringe pump was used to control the rate (mL/min) at which the inoculum in the syringe was added to water fed into the 7000RMS. To increase the concentration of beads/microorganisms added, the injection rate was increased on the syringe pump; conversely, to decrease the concentration, the syringe pump’s injection rate was decreased. Testing proceeded from lowest to highest injection rate. To limit variability between replicate results, injection rates were 25 µL/min or higher. Injection rates used for testing are listed in Table 7.

**Table 7.** Injection rates and concentrations tested for microorganisms

	Injection rate, 25 µL/min	Injection rate, 50 µL/min	Injection rate, 250 µL/min	Injection rate, 500 µL/min	Injection rate, 2500 µL/min	Injection rate, 5000 µL/min
Concentration, CFU/10 mL	50	100	500	1000	5000	10 000
Concentration, CFU/100 mL	500	1000	5000	10 000	Not tested	Not tested

In these experiments, the waste port outlet on the 7000RMS was used to collect a sample for testing by the traditional MF method. Water was temporarily diverted to allow for sanitization of the port with 6% H_2_O_2_ for a 10-minute contact time. Subsequently, an autoclaved collection line was installed on the port, and the free end of this line ran to a sterile bottle. After the line was installed, water was allowed to feed the 7000RMS once more creating a pathway for sample collection where the line could be transferred from the catch bottle to a sample container and then back again without interruption to the testing operation.

**Figure 3.**
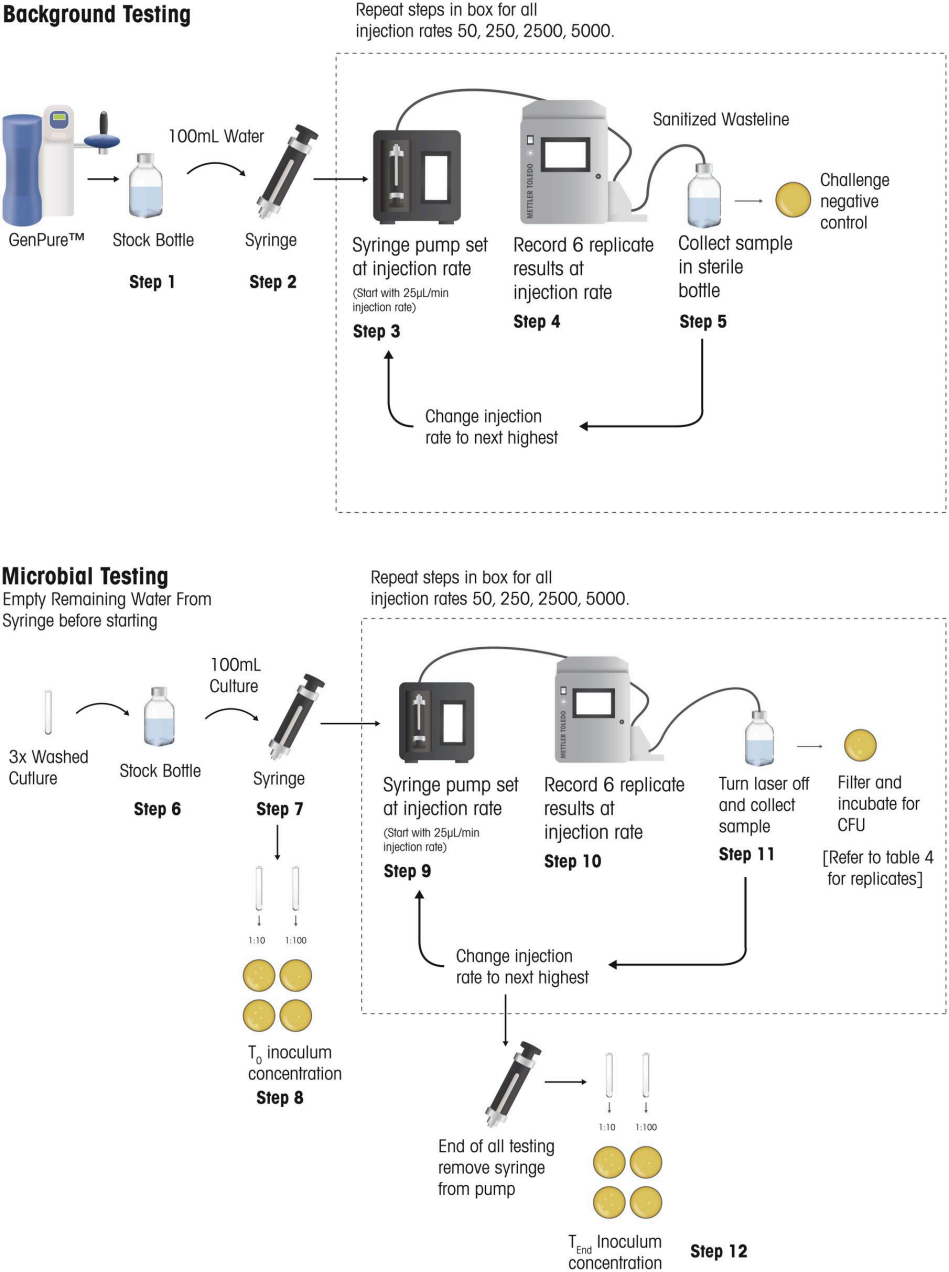
Microorganism testing.

Each microorganism listed in Table 2 was tested at five concentrations (corresponding to five different injection rates). At each concentration, six replicate tests were performed by both the 7000RMS and the MF method. Figure 3 shows the steps performed for microorganism challenges. Each microorganism challenge experiment consisted of five steps: *(1)* background analysis, *(2)* preparing the inoculum, *(3)* testing by the 7000RMS, *(4)* testing by the MF method, and *(5)* inoculum control testing and determination of concentration.

Prior to and after each challenge, the sample flow pathways in the 7000RMS were sanitized using H_2_O_2_ delivered at a 6% (v/v) concentration for 10 minutes. These pathways were then rinsed with 300 mL GenPure water to remove any residual H_2_O_2_.

As particles are ubiquitous in the environment, an analysis of background (Background Testing in Figure 3) was used to account for the addition of extraneous particles to the challenge. The background testing was executed using identical injection rates and replicates as the microorganism tests that followed.

A bottle was filled with 400 mL water (Step 1) and 93 mL was transferred from the bottle to a syringe (Step 2). The syringe was connected to the 7000RMS injection line and secured to the syringe pump. The remaining volume of the water in the bottle was capped and retained to create the microorganism inoculum (Step 7).

The injection line was primed by setting the injection rate to 5 mL/min for a few seconds and then the syringe pump was set to 25 µL/min, the lowest volume in Table 7 (Step 3). After a settling period of no more than 3 min where the AFU counts were observed, a total of six replicate results were recorded for the 25 µL/min injection rate (Step 4). The syringe pump was then set to 50 µL/min, the next highest injection rate in Table 5. Testing was repeated (Steps 3–4), for all the planned injection rates in Table 5, moving from the lowest to the highest rate. After acquiring the results from the final injection rate, the laser was turned off and a sample was collected from the sanitized sample collection port. This sample was filtered and served as the negative control for the challenge (Step 5).

The microorganism test inoculum described in the sample preparation section was transferred to the syringe (Step 7). The syringe was then connected to the injection line on the 7000RMS and secured to the syringe pump (Step 8).

The injection line was primed by setting the injection rate to 5 mL/min for a few seconds and then the syringe pump was set to 25 µL/min, the lowest volume in Table 7 (Step 9). After a settling period of no more than 3 min where the AFU counts were observed, a total of six replicate results were recorded for the 25 µL/min injection rate (Step 10). After the collection of the 7000RMS results, the laser was turned off and a sample for MF testing was collected from the 7000RMS sanitized sample collection line for MF testing (Step 11).

The laser was turned back on, and the injection rate was slowed to 10 µL/min to re-establish the baseline. The syringe pump was then set to 50 µL/min, the next highest injection rate in Table 7. Testing was repeated (Steps 9–11) for all the planned injection rates in Table 7, moving from the lowest to the highest rate. For microorganisms, a corresponding set of MF tests was completed at each test concentration analyzed by the 7000RMS. These samples were taken from the waste port on the 7000RMS after the completion of the 7000RMS analysis. Each MF sample collected was tested immediately to ensure the 7000RMS results and the MF plate times were as close as possible. The MF method is limited to a count of No More Than (NMT) 200 CFU per membrane filter. Therefore, in addition to the 10 mL test volume, lower volumes (1 mL and 100 µL) were tested to ensure countable plates were achieved. These volumes are shown in Table 8.

**Table 8.** MF test volumes used for each challenge concentration

Challenge concentration	Plating volumes (six each)
50 CFU/10 mL or 500 CFU/100 mL	10 mL, 1 mL
100 CFU/10 mL or 1000 CFU/100 mL	10 mL, 1 mL
500 CFU/10 mL or 5000 CFU/100 mL	10 mL, 1 mL
1000 CFU/10 mL	10 mL, 1 mL
5000 CFU/10 mL	10 mL, 1 mL, 100 µL
10 000 CFU/10 mL	10 mL, 1 mL, 100 µL

The MF sample test volume was transferred to a sterile filter funnel containing a 0.45 µm gridded nitrocellulose ester membrane filter. Vacuum was applied to draw the sample through the membrane filter which was then aseptically placed onto R2A. The R2A plate was allowed to incubate for 5–7 days at 30–35°C. A total of six MF tests were performed for each volume in Table 8.

After incubation, colonies were counted on each plate. The result for the MF test was determined from the six replicate plates at volumes which were least dilute as well as countable. In cases where the countable plate was not 10 mL, CFU counts were multiplied by the appropriate factor of 10 to achieve the appropriate 7000RMS test volume before comparing the 7000RMS result. For example, when a 1 mL result was the countable result within a set of concentrations, the CFU counts were multiplied by 10 so that the CFU result could be represented as CFU/10 mL.

After the completion of the final 7000RMS analysis and the collection of the final MF sample, a minimum of 3 mL of the inoculum in the syringe was collected for determining the T_end_ inoculum concentration at the end of the analysis (Step 12). Testing of this sample is described in the sample preparation section.

After the incubation period, all inoculum plates were examined for purity and an identification was conducted to confirm the microorganism identity. The total CFU on each inoculum control plate was counted. Counts above 200 CFU were considered uncountable.

**Figure 4.**
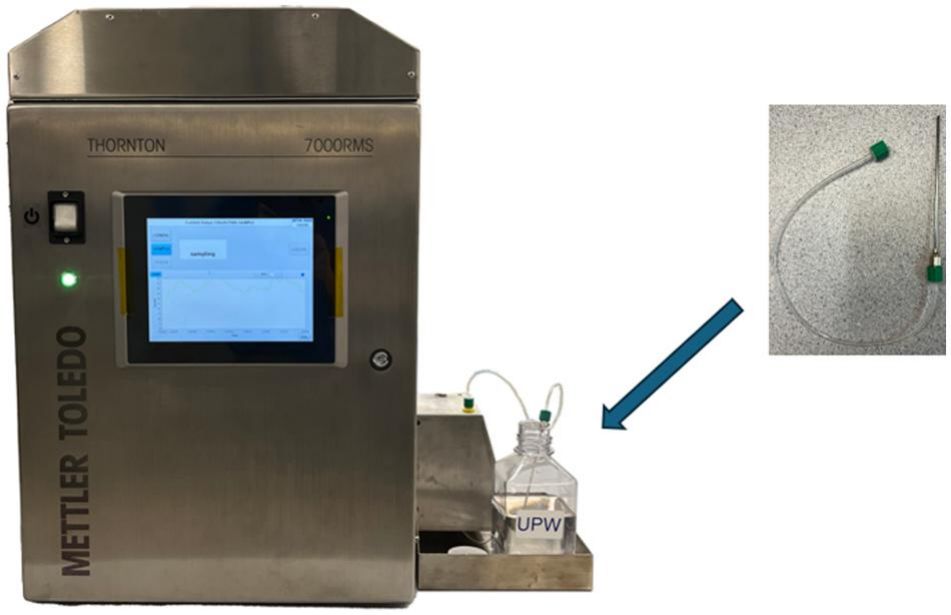
Sample mode configuration.

**(c)** *Bead testing.—*Experimental testing for beads using 7000RMS online mode was conducted similarly to microorganism challenges depicted in Figure 3 with the following exceptions:
  *(1)* The T_0_ inoculum concentration (Step 8), T_end_ inoculum concentration (Step 12) and MF testing (Step 11) were not performed as these steps were specific for microorganisms.
  *(2)* The negative control collected from the highest injection rate of the background analysis (Step 5) was omitted and instead a negative control was collected from the highest (and final) injection rate for bead testing (Step 11). The sample volume (60 mL for 10 mL test volumes and 100 mL for 100 mL test volumes) was passed through a 0.45 µm gridded cellulose-ester membrane filter with the aid of a vacuum pump. The filter was transferred to R2A and allowed to incubate for 5–7 days at 30–35°C.
  *(3)* Modifications to the injection rates and inoculum concentrations were made to accommodate a greater number of test concentrations across the range. Refer to Table 9.
  *(4)* No corresponding MF test was conducted; therefore, 7000RMS results were compared back to the calculated bead concentrations.
**(d)**  *Sample mode.—*Unlike testing conducted in online mode, the 7000RMS system operated with no additional equipment. A sample input tube with connectors and a sample syringe needle provided by the manufacturer with the purchase of the instrument was sterilized by autoclaving prior to each use and is shown in Figure 4. The sample input tube with connectors was screwed to the 7000RMS inlet port and the sample syringe needle was placed into the sample bottle. When the analysis is started on the 7000RMS, its internal pump draws the pre-defined volume of sample through the sample input tube with connector and sample syringe needle and into the 7000RMS system for analysis.

**Table 9.** Injection rates and concentrations tested for beads using online mode

10 mL testing	Inoculum concn, beads/mL	6 × 10^3^								
	Injection rate, µL/min	25	50	250	500	2500	5000	—^a^	—	—
	Concentration, beads/10 mL	50	100	500	1000	5000	10 000	—	—	—
10 mL testing	Inoculum concn, beads/mL	6 × 10^2^								
	Injection rate, µL/min	25	50	250	500	2500	5000	—	—	—
	Concentration, beads/10 mL	5	10	50	100	500	1000	—	—	—
100 mL testing	Inoculum concn, beads/mL	2 × 10^5^								
	Injection rate, µL/min	50	100	150	250	350	500	—	—	—
	Concentration, beads/10 mL	5	10	15	25	35	50	—	—	—
100 mL testing	Inoculum concn, beads/mL	3.3 × 10^5^								
	Injection rate, µL/min	5	10	15	25	35	50	250	500	1000
	Concentration, beads/10 mL	5	10	15	25	35	50	250	500	1000
100 mL testing	Inoculum concn, beads/mL	3.3 × 10^6^								
	Injection rate, µL/min	25	50	250	500	1000	—	—	—	—
	Concentration, beads/10 mL	250	500	2500	5000	10 000	—	—	—	—

a — = Null Data Set.

Only beads were tested by sample mode. The concentration of beads used in the validation was determined from the bead manufacturer’s label claim. Using this information, appropriate volumes of the prepared bead inoculum were transferred to the test bottle such that the contents of the bottle were at the required concentration per test volume.

**Figure 5.**
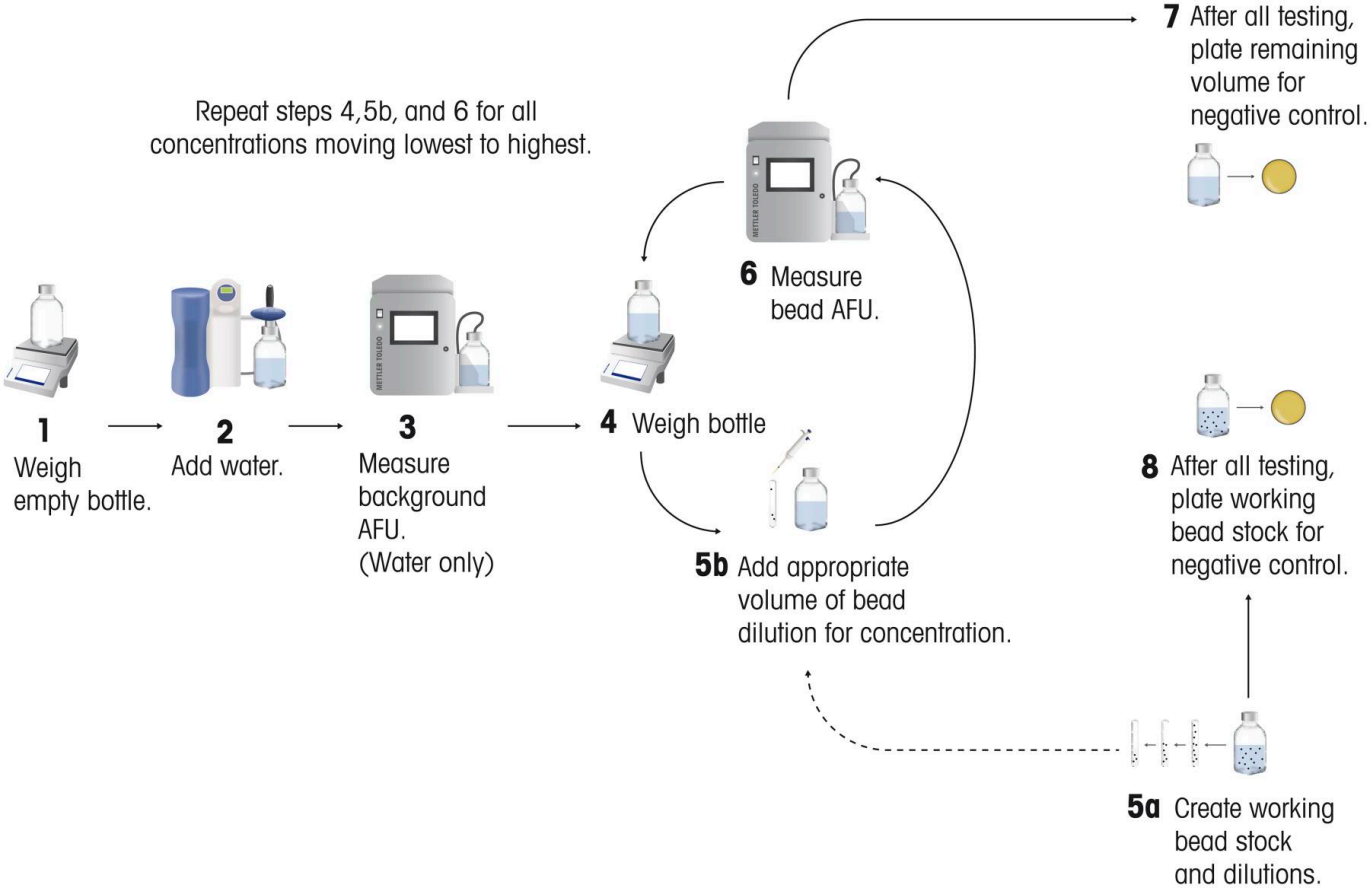
Sample mode testing of beads.

Figure 5 shows sample mode testing. Once started, the 7000RMS internal pump pulls the sample volumes through the system and each sample replicate is analyzed in a continuous manner following the prime volume.

A 2 L bottle was weighed (Step 1) and filled with water (Step 2). Just prior to analysis, the sample bottle was gently swirled to mix the contents before the straw was inserted into the bottle and secured to hang just slightly above the bottom. The 7000RMS was set to sample mode and a prime volume, a volume report interval (volume per replicate) and sample volume (total volume for all replicates) are entered. Once the analysis is started, the 7000RMS internal pump pulls the sample volumes through the system and each sample replicate is analyzed in a continuous manner following the prime volume. The first set of results were considered background as these were measurements of the water only (Step 3). Thirty replicate tests were recorded with 1 mL volumes. For 10 mL and 100 mL volumes, six replicate tests were recorded.

After the analysis of background, the bottle was weighed (Step 4), and the amount of water was determined by subtracting the original empty bottle weight from Step 1. The working bead stock solution was diluted in series to create several concentrations at 10^2^, 10^3^, and 10^5^ (Step 5a). The volume of beads needing to be added to the water to achieve the lowest concentration was determined using Equation (2), *seeCalculations*.

The beads were added to the bottle and gently mixed (Step 5 b). The bottle was then analyzed by the 7000RMS (Step 6). The number of recorded replicates was identical to the background testing (i.e., 30 or 6). Weighing (Step 4), bead addition (Step 5 b), and 7000RMS analysis (Step 6) were repeated, moving from the lowest to highest concentration until all concentrations were analyzed.

At the end of testing, a sample from the bottle (Step 7) and one from the bead working stock (Step 8) served as a control to confirm no extraneous microorganisms were in the test environment. Testing of this sample was performed by transferring it to a sterile filter funnel containing a 0.45 µm gridded nitrocellulose membrane filter. Vacuum was applied to draw the sample through the membrane filter which was then placed onto R2A. The R2A plate was allowed to incubate for 5–7 days at 30–35°C.

### Calculations

- *Microorganism inoculum concentration.—*For online mode testing, the T_0_ and T_end_ concentrations were independently calculated as the average of the least dilute duplicate plates divided by the dilution. The inoculum concentration was calculated as an average of the T_0_ and the T_end_ results.
- *Test concentration.—*For online mode testing, the test concentration at each injection rate was determined using the inoculum concentration and Equation (1), determination of test concentration at a specific injection rate. In all instances the 7000RMS flow rate was 30 mL/min:
  (1) $$\frac{\mathrm{Inoculum}\;\mathrm{concentration}\;\left(\frac{\mathrm{CFU}}{mL}\right)\cdot \;\mathrm{injection}\;\mathrm{rate}\;\left(\frac{mL}{\min}\right)}{7000RMS\;\mathrm{Flow}\;\mathrm{Rate}\;\left(\frac{mL}{\min}\right)}\cdot \mathrm{challenge}\;\mathrm{test}\;\mathrm{volume}\;\left(i.e.,\;10\;mL\;or\;100\;mL\right)$$
- *Bead concentration for sample mode.—* Equation (2), determination of bead stock volume, was used to determine the volume of a given prepared bead dilution that needed to be added to the water to achieve the test concentration.
  (2) $$\mathrm{Bead}\;\mathrm{volume}\;\left(mL\right)\;to\;\mathrm{add}=\frac{V\;\left[D-C\right]}{S\;}$$
     where V = current volume in the bottle (mL); D = desired concentration (beads/mL); C = current concentration (beads/mL); and S = concentration of beads to be added (i.e., 10^2^, 10^3^ or 10^5^ beads/mL)
     The following calculations were used for both online and sample mode testing.
- *7000RMS adjusted count.—*
7000RMS adjusted count=Geometric Mean of background AFU at rate n- reported AFU at rate 
     This calculation was performed for online and sample mode testing. The 7000RMS results from each injection rate (*n*) were adjusted when direct numerical comparisons between the reported AFU and the number of particles or microorganisms present were made. Such was the case for the accuracy validation parameter and, by extension, also LOQ, range, and equivalency, which required accuracy for their determination.
- *Detection scoring for 7000RMS.—*To determine whether a result was considered detected, each AFU result (signal) was divided by the average of the six background results. The resulting quotient was evaluated as follows:
  $$\begin{array}{c} \text{Detected}=\text{quotient of}\;3.0\;\text{or greater}\\ \text{Not detected}=\text{quotient of}\;2.9\;\text{or less}. \end{array}$$
    The detection criteria for the 7000RMS was derived from ICH Q2 (R1; 10) which is reiterated in USP <1225> Validation of Compendial Procedures (8). These documents establish an acceptable level of detection when “…acceptable signal to noise ratio is either 2:1 or 3:1” (8).
- *Percent recovery.—*When comparing the results of the 7000RMS to MF method for accuracy, the percent recovery was calculated following USP <61> as follows:
  $$\%\;\mathrm{Recovery}=\left(\mathrm{Average}\;\mathrm{Adjusted}\;\mathrm{RMS}\;\mathrm{Count}÷\mathrm{Average}\;MF\;\mathrm{Count}\right)\times 100\%$$
  When comparing the results of the 7000RMS or MF method the inoculum control counts for accuracy, the percent recovery was calculated as follows:
  % Recovery=(Average Count of the Method          (7000RMS Adjusted Count or MF Count)   ÷ Inoculum Count)×100%
- *Coefficient of variation percentage (%CV).—*When determining precision, the results of replicate testing of the same test concentration were used to determine %CV using the following equation:
  $$\%CV=[\mathrm{Standard}\;\mathrm{Deviation}\;\left(n_{1},\;n_{2\;},\;n_{3}\ldots n_{x}\right)÷\mathrm{Mean}\;\left(n_{1},\;n_{2\;},\;n_{3}\ldots n_{x}\right)]\;\times 100\%$$

### Validation Parameter Analysis

The validation was designed to follow the guidance in USP <1223> and EP Chapter 5.1.6 and PDA Technical Report 33 (1–3). The 7000RMS is considered a quantitative method as it provides numerical results. As such, accuracy, specificity, precision, LOD, LOQ, linearity, and range apply. Further, analysis of the validation criteria demonstrates performance equivalence testing per USP <1223> and a lack of correlation to the CFU.

### Accuracy Testing

Guidance documents define accuracy as the closeness of the actual test result obtained by the alternative test method to the value obtained by compendial method, demonstrated across the range. Due to the non-equivalence of the AFU to the CFU, direct comparisons of the numerical counts is impractical (5). Therefore, the experimental design developed was a multi-pronged approach using microorganisms and beads.

- *Microorganisms.—*Testing was performed for 11 microorganisms. Refer to Table 2. Results were assessed for test concentrations of 500 CFU/10 mL and above as this corresponds to the alert limit for PW in terms of CFU.
    Numerical comparisons between the 7000RMS and the MF methods to each other and to the inoculum control were made. Additionally, a comparison of each method’s capability for detecting each test microorganism was made.
  1. *Numerical comparisons.—*No acceptance criteria were applied for numerical comparisons due to the non-equivalence of CFU and AFU.
  2. *7000RMS compared to MF.—*Although no acceptance criteria were applied due to non-equivalence of CFU and AFU where less than 50% recovery was observed, a single repeat was performed using a 100 mL volume for any microorganism to rule out homogeneity issues.
  3. *Comparison to the inoculum control.—*For this comparison, each test concentration and microorganism combination were considered an individual test. The percent recovery of the inoculum control was calculated for both the 7000RMS and the MF method for each test. A comparison of the total number of tests that resulted in ≥50% recovery of the inoculum was tallied for each method. The 7000RMS was determined to have superior, equivalent, or inferior recovery of the inoculum compared to the MF test as follows:*(1)* Superior: The number of tests where the 7000RMS has ≥50% recovery of the inoculum *is greater than* the MF.*(2)* Equivalent: The number of tests where the 7000RMS has ≥50% recovery of the inoculum 7000RMS is *equal* to the MF.*(3)* Inferior: The number of tests where the 7000RMS has ≥50% recovery of the inoculum 7000RMS *is less than* the MF.Only these results were reported. No acceptance criteria were applied due to non-equivalence of CFU and AFU.*(4)* *Detection comparisons.—*Detection was scored for each method. Detection determination for the 7000RMS is described in *Calculations*, while any CFU recovery on the MF method was considered detection.
          Acceptance criteria were fulfilled when 100% of all microorganisms were detectable with the 7000RMS using either the 10 mL test volume or the 100 mL test volume, whichever was greater.
- *Beads.—*The operational modes, sample volumes, and test concentrations are listed in Table 10. It should be noted that, for online mode testing, multiple syringes of the bead inoculum were necessary to achieve all target concentrations due to the limitations in the syringe capacity of 93 mL.
    For each mode, volume, and test concentration, the percent recoveries were calculated. Accuracy was considered acceptable when the recovery was calculated to be ≥50.00% at the following concentrations:*(1)* 1 mL: 50–10 000 beads.*(2)* 10 mL: 500–10 000 beads.*(3)* 100 mL: 5000–10 000 beads.

**Table 10.** Accuracy test plan for beads

Mode	Volume	Test	Target test concentrations per volume
Sample mode (bottle)	1 mL	1	<1, 5, 50, 500, 5000, and 10 000
Online (syringe)	10 mL	2	5, 10, 50, 100, 500, and 1000
		3	50, 100, 500, 1000, 5000, and 10 000
Online (syringe)	10 0mL	4, 5	5^a^, 10^a^, 15^a^, 25^a^, 35^a^, and 50^a^
		6	5^a^, 10^a^, 15^a^, 25^a^, 35^a^, 50^a^, 250, 500, and 1000
		7	250, 500, 2500, 5000, and 10 000

a Testing was conducted using two separate syringe preparations to cover all intended concentrations due to a limited syringe capacity.

The lowest concentration is proportionally equivalent to the current PW alert limit, whereas the highest concentration is consistent with the tested range.

### Specificity Testing

Guidance documents define specificity as the ability to detect a variety of challenge microorganisms specific to the technology. To satisfy this guidance, 13 different microorganisms listed in Table 2 were tested for specificity. This panel provided for a range of microorganisms including Gram-negative and Gram-positive microorganisms, rods and cocci, a mixed culture, a stressed microorganism, a heat-shocked Bacillus preparation and two different isolates of the same species.

Initial testing was performed at concentrations of approximately 100 CFU/10 mL. Where one or more of the results had no detection on the 7000RMS, the test was repeated at 1000 CFU/100 mL to rule out issues with homogeneity or elevated backgrounds.

For each microorganism test, a comparison of percent detection was made between the 7000RMS and the MF for each microorganism.

1. Superior: 7000RMS percent detection *is greater than* the MF percent detection.
2. Equivalent: 7000RMS percent detection *is equal to* the MF percent detection.
3. Inferior: 7000RMS percent detection *is less than* the MF percent detection.

The total number of superior, equivalent, and inferior were determined. Specificity was considered acceptable if the 7000RMS was equivalent or superior for the panel of microorganisms as often or more often than the MF method.

### Interferents/Suitability Testing

All samples tested by the 7000RMS system are intended to be PW or higher purities of water. As such, the sample types are not considered unique and suitability demonstration is not required during laboratory validation. Instead, interferent testing should be performed to demonstrate that the instrument installation or sample collection does not augment (false positive) or diminish (false negative) the AFU results.

Online mode interferent testing is reserved for in situ testing in the field and was not covered in the laboratory validation. However, since sample mode testing requires the collection of water in containers, interferent testing was completed in this laboratory validation using 2 L PTEG and glass containers. Each container type was tested twice, once at 1 mL and once at 10 mL volume.

This testing was performed based on guidance in EP <5.1.6> (2) for non-growth-based methods and PDA Technical Report 33 (3). Due to the volume restrictions of the containers, 30 replicates were acquired for the 1 mL test volume, and six replicates were acquired for 10 mL samples. Each bottle was filled with GenPure water and the contents of the container were tested by the 7000RMS. For each container, the highest AFU count was determined from all replicates.

After the AFU measurements of the water, beads at the LOD concentration of 25 beads per 100 mL were added to each bottle. For each container, the lowest AFU count was determined from all replicates. The lowest bead AFU count was divided by the highest water AFU count. The interferent test was considered acceptable where the quotient was calculated to be ≥3.0 for all trials.

### PrecisionTesting

Guidance documents define precision as the degree of agreement among individual test results when the procedure is applied repeatedly to multiple samplings of the same suspension and using different suspensions across the range of the test. Precision was demonstrated using beads, microorganisms, and both modes.

- *Beads.—*The capability for the 7000RMS to produce precise results in a variety of operational modes and volumes was challenged using a low-intensity fluorescent bead. The % CV was calculated at each concentration.
  1. Testing was conducted at 100 mL in online mode to represent the intended routine use where the instrument is installed on a pharmaceutical water loop. Acceptance criteria was set at ≤35% CV for concentrations ≥10 000 beads/100 mL.
  2. Testing was conducted at 1 mL in sample mode to represent the intended occasional testing from containers. Acceptance criteria was set at ≤35% CV for concentrations ≥100 beads/mL.
- *Microorganisms.—*A total of nine different microorganisms were tested (refer to Table 2) and % CV was evaluated at each test concentration. Acceptance criteria was set as 70% of all microorganism challenges result in ≤35% CV at or above test concentrations of 500 CFU/10 mL.

A 35% CV was applied based on guidance from both USP <1223> and PDA Technical Report 33, which indicate that the precision criteria in the range of 15–35% is reasonable for a microbiological method.

The number of challenges required to meet the %CV criteria was set at 70%; however, the concentration threshold was set lower at the alert limit (500 CFU/10 mL), rather than the higher action limit (1000 CFU/10 mL). The criteria were selected to account for any imprecision related to the injection process itself, which is a continuous addition in a stream of water rather than replicate results tested from a single sample container as with the traditional MF test.

### LOD Testing

Guidance documents define the LOD as the lowest number of microorganisms in a sample that can be detected, but not necessarily quantified. The detection capabilities of each method were compared to each other as suggested by PDA Technical Report 33. Additionally, beads were used to determine the LOD value itself.

- *Beads.—*Both online testing using a syringe injection and at-line testing as indicated in Table 11. The aim of each mode/concentration was different.
  1. *Sample mode.—*A 1 mL volume was utilized as testing larger volumes in a single container proved ergonomically prohibitive. Testing in sample mode, while not identical to the online mode, aids in determining where the LOD may be established. Two challenges were completed on separate days.
  2. *Online mode.—*A 100 mL volume was tested to be consistent with the intended use. While testing at lower concentrations (5–50 beads/100 mL) had a total of three challenges to establish an LOD value, testing concentrations of 250 beads/100 mL and above served as additional confirmation that consistent detection was achieved throughout the range.
     For each challenge, the replicate results at each test concentration were scored for detection. The detection percentage was calculated for that challenge concentration. The results from all challenges were combined and ordered by concentration to determine the lowest concentration where there was 100% detection for all challenges. The LOD was established at this point. The established LOD value was considered acceptable if it was no worse than 5000 counts/100 mL (or equivalent concentration of 50 beads per 1 mL). The LOD criteria was set at the established CFU alert level for PW water.
- *Microorganisms.—*Each replicate result from all challenge concentrations equal to or greater than the internal water alert level (500 CFU/10 mL or 5000 CFU/100 mL) was scored as “detected” or “not detected”. The detection outcomes for all 12 microorganisms tested were summed and a detection percentage for both the MF and the 7000RMS was determined. This comparison was considered acceptable if the number of tests detected by the 7000RMS was equivalent or greater than that of the MF method. This acceptance criteria was derived from PDA Technical Report 33 which states, “In general, the limit of detection of the alternative or rapid method should not be significantly worse than that of the existing method.”

**Table 11.** LOD bead testing plan

Operational mode	Test volume	Target concentration	Number of replicates per concentration	Number of challenges
Sample	1 mL	<1, 5, 50, 500, 5000, and 10 000	30	2 (different days)
Online	100 mL	5, 10, 15, 25, 35, and 50	6	3 (different days)
		250 and 500	6	2
		1000, 2500, 5000, and 10 000	6	1

### LOQ Testing

LOQ is the lowest number of microorganisms in a test sample that can be enumerated with acceptable accuracy and precision. Due to the non-equivalency of the AFU to the CFU, the LOQ for the 7000RMS was determined by beads using a 100 mL volume tested using both operational modes. Concentrations spanning both below and above the presumed LOQ value were challenged. Three separate tests were performed on separate test dates, each using a unique bead preparation. Each challenge was assessed as to whether the results were accurate (% recovery is ≥50%), and precise (%CV is ≥35%).

The LOQ value was determined from the lowest concentration where all three tests were accurate and precise. The established LOQ was considered acceptable if the value was worse than 100 000 counts/100 mL. This LOQ criteria was set at an equivalent concentration to the 100 CFU/mL established CFU action level for PW water.

### Linearity Testing

Guidance documents define linearity as the ability to produce results that are proportional to the concentration of microorganisms present in the sample within a given range.

Linearity was assessed using 11 microorganisms and one low-intensity fluorescent bead as listed in Table 2. Testing consisted of multiple test concentrations and both operation modes. These are summarized in Table 12. A linear regression analysis was performed using the calculated CFU test injection concentration as the independent or x-variable and the unadjusted 7000RMS AFU count as the response or y-variable.

**Table 12.** Test plan for linearity

Analyte	Target concentrations	Volume tested	Replicates per concentration	Number of challenges	Operational mode
Microorganisms	50, 100, 500, 1000, 5000, and 10 000 CFU	10mL	6	1–2	Online
Beads	Syringe 1^a^: 25, 50, 100, 500, and 1000 beads	100 mL	6	1	Online
	Syringe 2^a^: 250, 500, 2500, 5000, and 10 000 beads				
	Syringe 1^a^: 5, 10, 50, 100, 500, and 1000 beads	10 mL	6	1	Online
	Syringe 2^a^: 50, 100, 500, 1000, 5000 and 10 000 beads				
	Each bottle: 5, 10, 50, 100, 500, 1000, 5000 and 10 000 beads	1 mL	30	2	Sample

a Testing was conducted using two separate syringe preparations to cover all intended concentrations due to a limited syringe capacity.

**Table 13.** Validation parameter acceptance criteria and results

Performance characteristic	Acceptance criteria	Results	Met/ Not met
Accuracy	Microorganism challenges		Met
	Comparisons: (Informational, no criteria due to non-equivalence of CFU and AFU.) Method to method: The 7000RMS AFU count is NLT^a^ 50% of MF CFU for all microorganisms. Method to inoculum control: The 7000RMS has equivalent or superior recovery of the inoculum control than the MF method for all microorganisms. Detection: The 7000RMS detects all microorganisms.	Comparisons: (Results presented for informational purposes only) Method to method comparison: 7000RMS has 50% or greater recovery for 5 of 12 (42%) microorganisms. Method to inoculum control comparison: The 7000RMS had equivalent or superior recovery of the inoculum compared to the MF method for 5 of 12 (42%) microorganisms Detection: 12 of 12 (100%)	
	Bead challenges		
	The 7000RMS shall recover ≥50.00% of the theoretical bead concentration when tested at defined concentration intervals using the following volumes: 1 mL: 50–10 000 beads 10 mL: 500–10 000 beads 100 mL: 5000–10 000 beads	The 7000RMS recovered ≥50.00% of the theoretical bead concentrations when tested at defined concentration intervals using the following volumes: 1 mL: ≤1–10 106 beads 10 mL: 5–10 134 beads 100 mL: 15–10 030 beads	
Interferent/suitability	There are no interfering factors in water samples.	There are no interfering factors in water samples.	Met
Specificity	The 7000RMS was able to detect all microorganisms as often or better than the MF method.	The 7000RMS was able to detect all microorganisms as often or better than the MF method.	Met
LOD	Microorganism challenges		Met
	The percentage of organisms detected by the 7000RMS shall be greater than or equal to that of the MF method.	The 7000RMS can detect a greater percentage of microorganisms compared to the MF method.	
	Bead challenges		
	5000 beads per 100 mL or better (i.e., lower).	25 counts per 100 mL	
LOQ	10 000 beads per 100 mL or better (i.e., lower).	25 counts per 100 mL	Met
Linearity	R^2^ value of 0.9025 or better (i.e., higher).	R^2^ value of 0.9077 or higher was achieved in all test cases.	Met
Precision	Microorganism challenges		Met
	At least 70% of tests have a %CV of ≤35% at concentrations greater than 500 CFU/10 mL (or equivalent concentration of 5000 beads per 100 mL).	100% of microorganism tests have %CV of ≤35%.	
	Bead challenges		
	%CV is ≤35% for concentrations greater or equal to 100 beads/mL or 10 000 beads per 100 mL.	%CV is ≤35% at concentrations greater than or equal to 50 beads/mL and 25 beads/100 mL.	
Range	Lower bound: LOQ Upper bound: NLT^b^ 10 000 counts per 100 mL	Lower bound: 25 counts per 100 mL (LOQ) Upper bound: 10 030 counts per 100 mL	Met
Equivalency	The 7000RMS shall have equivalent or better overall performance than MF method for at least three of the four parameters specificity, accuracy, precision, and LOD	The 7000RMS showed equivalent or better performance than MF for at least three of the four specified parameters: Specificity—equivalent^b^ Precision—equivalent^b^ LOD—superior^b^ Accuracy—inferior^b^	Met

a NLT =  Not less than.

b Equivalent, superior and inferior are total number comparisons and not statistical.

To ensure that linearity was not influenced by variability in the microorganism inoculum, microorganisms that had a greater than 1 log change between the T_0_ and T_end_ inoculum count were not assessed for linearity as this change in viability was indicative of the inability to culture the microorganism ((e.g., Viable but Nonculturable (VBNC)) rather than a true assessment of 7000RMS linearity.

To align with recommendations in USP <1223> and PDA Technical Report 33, the acceptance criteria for linearity was a calculated R^2^ value of ≥0.9025 applied to the following results (1, 3).

1. Microorganisms: average of all challenges.
2. Beads (bottle): both challenge preparations and combined data from both dates.
3. Beads (syringe, 10 mL): each challenge and combined data for the volume.
4. Beads (syringe, 100 mL): each challenge and combined data for the volume.

### Operational Range and Equivalence Testing

The operational range is defined by guidance documents as the interval between the upper and lower levels of microorganism that have been demonstrated to be determined with accuracy, precision, and linearity. Data from beads tested at the 100 mL volume using online mode were used to determine the operational range as this was consistent with the user intent for monitoring online continuously.

Performance equivalence per USP <1223> allows for a comparison of results between the current and alternative methods (1). Microorganism results for accuracy, specificity, precision, and LOD were evaluated and the performance of the 7000RMS was compared to the MF method. The 7000RMS was considered equivalent to the MF method where three of the four parameters had equivalent or superior performance.

### Correlation Testing

Data from the microorganism testing results were analyzed for correlation. All replicate results from MF and 7000RMS results at all concentrations from 10 mL testing were used. The correlation analysis was conducted using both transformed log 10 fits and untransformed fits. A one-way analysis of variance (ANOVA) was performed to determine if there was a statistically significant difference in the microorganism slope and y-intercepts result. There were no predetermined acceptance criteria as guidance documents recognize that correlation between methods is not always possible. A single correlation factor could not be established for comparing AFU and historical CFU data.

## Results and Discussion

The 7000RMS met all predetermined laboratory validation acceptance criteria. Table 11 summarizes the results and acceptance criteria for each performance characteristic. Each performance characteristic is discussed in more detail in subsections titled with the validation parameter.

### Accuracy

Results for the microorganism numerical comparisons and detection are in Table 14. In a comparison of the CFU results of the AFU results from the 7000RMS tests to the CFU counts of the MF test, 5 of 12 (42%) microorganisms had a calculated recovery of 50% or greater. Since the inoculum control was determined by MF, it is also no surprise that the comparison of results of each method to the inoculum control CFU shows that the same five microorganisms have equivalent or superior recovery with the 7000RMS. There is no pattern of recovery results that align with conventional characteristics such as size, shape, or Gram stain reaction of microorganisms. The detection comparison acceptance criteria were met since 12 of 12 (100%) of all microorganisms tested were detectable.

**Table 14.** Percent recovery and detectability results—microorganisms

Microorganism	Shape	Gram stain	≥50.00% Recovery of MF Method	Recovery of the inoculum	Detectable on 7000RMS
*P. paraeruginosa*	Rod	Negative	Yes	Equivalent	Yes
*B. spizizenii*	Rod	Positive	Yes	Superior	Yes
*B. spizizenii* (heat-shocked)	Spore/rod	Positive	Yes	Equivalent	Yes
*R. pickettii* (in-house strain No. 1)	Rod	Negative	No	Inferior	Yes
*R. pickettii* (in-house strain No. 2)	Rod	Negative	No	Inferior	Yes
*S. aureus*	Cocci	Positive	No	Inferior	Yes
*S. maltophilia*	Rod	Negative	No	Inferior	Yes
*P. fluorescence* (starved)	Rod	Negative	Yes	Equivalent	Yes
*P. glucanolyticus*	Rod	Positive	Yes	Superior	Yes
*E. coli*	Rod	Negative	No	Inferior	Yes
*S. paucimobilis*	Rod	Negative	No	Inferior	Yes
*B. cepacia*	Rod	Negative	No	Inferior	Yes

Interestingly, when examining the LOD results for these microorganisms (Tables 15 and 16), there is superior recovery of inoculum counts by the 7000RMS for *B. spizizenii,* and *P. glucanolyticus.* The results show that the MF test was unable to recover CFU in many of the tests, whereas the 7000RMS had AFU counts that increased as expected in proportion to the inoculum concentration (injection rate).

**Table 15.** *B. spizizenii* LOD comparison of 7000RMS to the MF method

Inoculum injection rate	Inoculum concn per 10 mL	Test method	Test replicate result (Yes = detected, No = not detected)						Total replicates detected	Method average count per 10 mL
			1	2	3	4	5	6		
25 µL	7	7000RMS	Yes	Yes	Yes	Yes	Yes	Yes	6	490
		Plate count	No	Yes	No	No	No	Yes	2	0.3
50 µL	14	7000RMS	Yes	Yes	Yes	Yes	Yes	Yes	6	1434
		Plate count	No	Yes	Yes	No	Yes	Yes	4	0.8
250 µL	71	7000RMS	Yes	Yes	Yes	Yes	Yes	Yes	6	6162
		Plate count	No	Yes	Yes	Yes	Yes	Yes	5	2
500 µL	142	7000RMS	Yes	Yes	Yes	Yes	Yes	Yes	6	12 656
		Plate count	Yes	Yes	Yes	Yes	Yes	No	5	2
2.5 mL	708	7000RMS	Yes	Yes	Yes	Yes	Yes	Yes	6	54 491
		Plate count	Yes	Yes	Yes	Yes	No	No	4	2
5.0 mL	1417	7000RMS	Yes	Yes	Yes	Yes	Yes	Yes	6	81 410
		Plate count	Yes	Yes	Yes	Yes	Yes	No	5	1

**Table 16.** *P. glucanolyticus* LOD comparison of 7000RMS to the MF method

Inoculum injection rate	Inoculum concn per 10 mL	Test method	Test replicate result (≥50% recovery of inoculum)						Total replicates detected	Method average count per 10 mL
			1	2	3	4	5	6		
25 µL	4	7000RMS	Yes	Yes	Yes	Yes	Yes	Yes	6	57
		Plate count	No	No	No	No	No	No	0	0
50 µL	8	7000RMS	Yes	Yes	Yes	Yes	Yes	Yes	6	125
		Plate count	No	No	No	No	No	No	0	0
250 µL	38	7000RMS	Yes	Yes	Yes	Yes	Yes	Yes	6	670
		Plate count	No	No	No	No	No	No	0	0
500 µL	75	7000RMS	Yes	Yes	Yes	Yes	Yes	Yes	6	1415
		Plate count	No	No	No	No	No	No	0	0
2.5 mL	375	7000RMS	Yes	Yes	Yes	Yes	Yes	Yes	6	7097
		Plate count	No	Yes	No	No	No	Yes	2	0.3
5.0 mL	750	7000RMS	Yes	Yes	Yes	Yes	Yes	Yes	6	13 641
		Plate count	Yes	No	No	No	Yes	No	2	0.3

The inoculum T_0_ concentrations were 1.7 × 10^3^ CFU/mL, and 9.2 × 10^2^ CFU/mL for *B. spizizenii,* and *P. glucanolyticus,* respectively. However, by the end of testing, the inoculum concentration declined significantly such that a change of 2–3 log was seen over the full testing period. These results were not surprising as method development data showed similar behaviors (data not provided). Despite the decrease in viable recovery, the 7000RMS continued to report counts in proportion to the increase in injection rate. These data suggest that that the 7000RMS can detect injured or otherwise VBNC microorganisms.

As previously discussed, a numerical comparison is problematic because an AFU is not equivalent to a CFU. The assessment of accuracy in terms of a direct numerical comparison is further compounded by examining the inaccuracies of the CFU itself. As stated in USP < 1223>, “It is important to understand that the CFU has always been an estimation of microorganisms present rather than an actual count.” This is because a CFU may be derived for one or more cells and its detection is based on the conditions present during its growth (e.g., temperatures and media; 1).

Determination of accuracy is more appropriately determined by a comparison of counts to an accurate standard. At this point in time there is no National Institute of Standards and Technology (NIST) traceable reference standard with appropriate size and fluorescence. The closest standard reference material, that is commercially available with the appropriate measured size and fluorescence is the yellow, low-intensity, 0.7–0.9 µm, fluorescent bead from Spherotech (Cat. No. FL-0852-2). Results met predetermined acceptance critera for the accuracy parameter as the 7000RMS could recover ≥50.00% of the theoretical bead count at all tested concentrations inclusive of the minimum and maximum concentrations as follows:

1. 1 mL: <1–10 106 beads.
2. 10 mL: 5–10 134 beads.
3. 100 mL: 15–10 030 beads.

### Specificity

Specificity results are summarized in Table 17. The 7000RMS was able to detect all microorganisms as often or better than the MF method. All 13 microorganisms resulted in 100% detection for all six replicate tests for the 7000RMS, while the MF method could only detect *B. spizizenii* 83% of the time and *P. glucanolyticus* 33% of the time.

**Table 17.** Specificity results

Microorganism	% Detection 7000RMS	% Detection MF Method	7000RMS comparison conclusion	Total
*P. paraeruginosa*	100	100	Equivalent	11
*B. spizizenii* (heat-shocked)	100	100	Equivalent	
*R. pickettii* (in-house strain No. 1)	100	100	Equivalent	
*R. pickettii* (in-house strain No. 2)	100	100	Equivalent	
*S. aureus*	100	100	Equivalent	
*S. maltophilia*	100	100	Equivalent	
*P. fluorescens* (starved)	100	100	Equivalent	
*E. coli*	100	100	Equivalent	
*S. paucimobilis*	100	100	Equivalent	
*B. cepacia*	100	100	Equivalent	
Mixed culture of *S. aureus* & *P. paraeruginosa*	100	100	Equivalent	
*B. spizizenii*	100	83	Superior	2

The inability of the MF method to consistently detect *B. spizizenii* and *P. glucanolyticus* is not unexpected. Per USP <1223>, “It is a normal characteristic of a conventional growth-based method to recover some species well and yet be unable to recover others.” (1). Further, per USP <1223>, “Studies on the recovery of microorganisms from potable and environmental waters have demonstrated that traditional plate-count methods reporting cell count estimates as CFUs may recover 0.1%–1% of the actual microbial cells present in a sample.” (1). Interestingly, for *B. spizizenii*, the heat-shocked microorganism resulted in recovery from all six replicates (100% recovery). As a heat shocking of *B. spizizenii* will cause the microorganism to produce spores, it is likely that the predominance of spores in this heat-shocked preparation compared to the non-heat-shocked preparation may be the difference in the *B. spizizenii* recovery on the MF plates. No such difference is seen between the 7000RMS results for *B. spizizenii* suggesting that the 7000RMS can detect both spores and vegetative cells.

### Interferent/Suitability

The PETG and glass bottles resulted in no interference at the LOD of 25 counts and above. These data indicate that neither the PW sample type nor the container material (i.e., PETG or glass) interfere with the 7000RMS detection. As previously discussed, this laboratory test does not demonstrate the necessary system suitability for instrument installation in the user water system location. Additional testing is required during performance qualification to demonstrate that the in situ use conditions remain acceptable.

### Precision

The 7000RMS was found to provide precise results for all nine of the microorganisms tested. Replicate results when testing the same concentration six times had a ≤35% CV at concentrations of 500 CFU/10 mL. Five of the microorganisms resulted in acceptable %CVs at concentrations below 500 CFU/10 mL (refer to Table 18).

**Table 18.** Precision results for microorganisms^a^^,^^b^

Microorganism		Results for target concentration per 10 mL					
		50	100	500	1000^b^	5000^b^	10 000
*P. aeruginosa*	Actual concn	48	95^a^	475^a^	950	4750	9500^b^
	Precision	Not met	Met^a^	Met^a^	Met	Met	Met^b^
*B. spizizenii* (heat-shocked)	Actual concn	34^a^	68^a^	342^a^	683	3417	6833^b^
	Precision	Met^a^	Met^a^	Met^a^	Met	Met	Met^b^
*R. pickettii* (strain No. 1)	Actual concn	34	68^a^	342	683	3417	6833^b^
	Precision	Not met	Met^a^	Not met	Met	Met	Met^b^
*S. aureus*	Actual concn	32	63	317	633	3167	6333^b^
	Precision	Not met	Not met	Not met	Met	Met	Met^b^
*S. maltophilia*	Actual concn	50	100	500	1000	5000	10 000^b^
	Precision	Not met	Not met	Met	Met	Met	Met^b^
*P. fluorescence* (starved)	Actual concn	32^a^	158^a^	317^a^	1583	3167	—^c^
	Precision	Met^a^	Met^a^	Met^a^	Met	Met	
*E. coli*	Actual concn	—	92	184^a^	921	1842	9208^b^
	Precision		Not met	Met^a^	Met	Met	Met^b^
*S. paucimobilis*	Actual concn	30	150	300^a^	1500	3000	—
	Precision	Not met	Not met	Met^a^	Met	Met	
*B. cepacia*	Actual concn	44	88^a^	442^a^	883	4417	8833^b^
	Precision	Not met	Met^a^	Met^a^	Met	Met	Met^b^

a Precision is met below required concentration of 500 CFU per 10 mL

b Precision at or above required concentration of 500 CFU per 10 mL

c — = Null Data Set.

The 7000RMS bead testing results were also precise. Results from two or more trials, using different sample volumes and both operational modes, show that replicate results have a 35% CV or less.

When testing beads using a 1 mL volume in sample mode, all concentrations at 50 beads or greater showed ≤35% CV between the 30 replicate tests (Table 19). The first trial resulted in ≤35% CV at all concentrations, whereas the second trial had higher imprecision at concentrations of <1 and 5 beads/mL. Since both trials resulted in ≤35% CV at 50 beads/mL, these data met the predetermined acceptance criteria of ≤35% CV at 100 beads/mL and greater.

**Table 19.** Precision—bead testing at 1 mL by sample bottle

Test		Results for target concentration 1 mL, sample bottle					
		<1	5	50	500	5000	10 000
1	Actual concn	<1	5	50	500	5003	10 105
	Precision	Not met	Not met	Met	Met	Met	Met
2	Actual concn	<1	5	50	582	5033	10 032
	Precision	Met	Met	Met	Met	Met	Met

When testing beads using a 100 mL volume in online mode, concentrations at 25 beads or greater showed ≤35% CV between the six replicate tests (Table 20). All three trials showed the same precision. Concentrations at 5, 10, and 15 beads/100 mL did not achieve ≤35% CV, whereas concentrations of 25 beads/100 mL and greater met the predetermined acceptance criteria of ≤35% CV at 10 000 beads/100 mL and greater.

**Table 20.** Precision—bead testing at 100 mL by injection

Test		Results for target concentration 100 mL, injection											
		5	10	15	25	35	50	250	500	1000	2500	5000	10 000
1	Actual concn	5	10	15	25	35	50	—^a^	—	—	—	—	—
	Precision	Not met	Not met	Not met	Met	Met	Met	—	—	—	—	—	—
2	Actual concn	5	10	15	25	35	50	—	—	—	—	—	—
	Precision	Not met	Not met	Not met	Met	Met	Met	—	—	—	—	—	—
3	Actual concn	5	10	15	25	35	50	251	503	1006	—	—	—
	Precision	Not met	Not met	Not met	Met	Met	Met	Met	Met	Met	—	—	—
4	Actual concn	—	—	—	—	—	—	251	501	—	2507	5015	10 030
	Precision	—	—	—	—	—	—	Met	Met	—	Met	Met	Met

a — = Null Data Set.

The bead precision results are used to establish LOD and LOQ for the 7000RMS. However, it should be noted that the precision determined by this validation is likely lower than these results show. Precision at extremely low levels is very difficult due to the inherent imprecision with laboratory measurements and the lack of a particle-free environment. While a greater number of replicates may have reduced some of the inherent imprecision, it is neither possible nor practical to do so given the amounts of sample materials and time it would have taken to do so. Further, it is unlikely that precision can be experimentally demonstrated at very low numbers as the principles of the Poisson distribution suggest that the percentage of error increases as the number of counts decreases (1).

### LOD

Testing of beads established the LOD at 25 counts/100 mL and data are summarized in Table 21. This LOD exceeded the acceptance criteria of 5000 counts/100 mL (or equivalent or equivalent concentration of 50 beads per 1 mL) by 200-fold. However, it is likely that the LOD is lower than what was found through this testing. That is, as the concentrations decrease, laboratory conditions must provide for testing conditions where all water (background) testing is virtually zero AFU. A background level that approaches zero is extremely difficult to achieve on a consistent basis as particles are ubiquitous in the laboratory environment. This is recognized even in particulate matter in injections testing per harmonized USP <788>, which allows for not more than 25 particles in 25 mL for the environment to be considered suitable for testing (11).

**Table 21.** LOD results for beads

Concentration	Mode	Challenge No.	Volume	% Detection
<1	At-line	1	1mL	97
	At-line	2	1 mL	100
5	At-line	1	1 mL	90
	At-line	2	1 mL	100
	Online	1	100 mL	17
	Online	2	100 mL	83
	Online	3	100 mL	33
10	Online	1	100 mL	50
	Online	2	100 mL	100
	Online	3	100 mL	0
15	Online	1	100 mL	83
	Online	2	100 mL	100
	Online	3	100 mL	33
25	LOD			
25	Online	1	100 mL	100
	Online	2	100 mL	100
	Online	3	100 mL	100
35	Online	1	100 mL	100
	Online	2	100 mL	100
	Online	3	100 mL	100
50	At-line	1	1 mL	100
	At-line	2	1 mL	100
	Online	1	100 mL	100
	Online	2	100 mL	100
	Online	3	100 mL	100
250	Online	1	100 mL	100
	Online	2	100 mL	100
500	At-line	1	1 mL	100
	At-line	2	1 mL	100
	Online	1	100 mL	100
	Online	2	100 mL	100
1000	Online	1	100 mL	100
2500	Online	1	100 mL	100
5000	At-line	1	1 mL	100
	At-line	2	1 mL	100
	Online	1	100 mL	100
10 000	At-line	1	1 mL	100
	At-line	2	1 mL	100
	Online	1	100 mL	100

The detection capabilities of the 7000RMS and MF method were compared using 12 different microorganisms. *See*Table 22. Replicate testing for all microorganisms where the inoculum concentrations were ≥500 CFU/10 mL (or equivalent concentration of 5000 CFU/100 mL) were scored as “detected” or “not detected”. The results met the predetermined acceptance limits as the number of tests detected by the 7000RMS was greater than that of the MF method. Out of 204 total tests, the 7000RMS was able to detect all 204 (100%), whereas the MF method was able to detect 197 (97%). Consistent with other validation parameters, the MF method fell short on detecting *P. glucanolyticus* and *B. spizizenii,* as only 2 of 6 and 9 of 12 tests were detected (i.e., count ≥1 CFU/10 mL), respectively.

**Table 22.** LOD for microorganisms

Microorganism	Total tests	7000RMS total “Detected”	MF method total “Detected”
*P. paraeruginosa*	18	18	18
*B. spizizenii*	12	12	9
*B. spizizenii* (heat-shocked)	18	18	18
*R. pickettii* (strain No. 1)	18	18	18
*S. aureus*	18	18	18
*S. maltophilia*	24	24	24
*P. fluorescens* (starved)	12	12	12
*P. glucanolyticus*	6	6	2
*E. coli*	24	24	24
*S. paucimobilis*	12	12	12
*B. cepacia*	18	18	18
Mixed culture (*P. paraeruginosa* and *S. aureus*)	24	24	24
Total tests with detection	204	204	197

### LOQ

Data from precision and accuracy testing of beads at 100 mL using the online method were complied. The LOQ for the 7000RMS was determined to be 25 counts/100 mL. The results for the LOQ testing are shown in Table 23. The LOQ was constrained by precision as the lower concentrations of beads at 5, 10, and 15 counts/100 mL resulted in a %CV above 35%. As discussed for the precision validation parameter, it is not likely that laboratory testing can demonstrate the actual capabilities of the 7000RMS. A consideration for demonstrating LOQ in the future is a comparison of %CV calculated for the MF method and the 7000RMS method to show that the 7000RMS is at least as precise as the MF method.

**Table 23.** LOQ results

Concentration, beads/100 mL	Test No. 1			Test No. 2			Test No. 3		
	Accurate	Precise	Outcome	Accuracy	Precision	Outcome	Accuracy	Precision	Outcome
5	Yes	No	Not met	Yes	No	Not met	No	No	Not met
10	Yes	No	Not met	Yes	No	Not met	No	No	Not met
15	Yes	No	Not met	Yes	No	Not met	Yes	No	Not met
25^a^	Yes	Yes	Met	Yes	Yes	Met	Yes	Yes	Met
35	Yes	Yes	Met	Yes	Yes	Met	Yes	Yes	Met
50	Yes	Yes	Met	Yes	Yes	Met	Yes	Yes	Met

### Linearity

When determining linearity, the data are determined to be more linear the closer an R^2^ value is to 1.000. Per guidance documents, microbiological test results collected over the range of the method that have a calculated R^2^ value of ≥0.9025 are considered acceptably linear. The assessment of linearity for the 7000RMS was determined using both microorganisms and beads.

A summary of all microorganism linearity results can be found in Table 24. The 7000RMS passed acceptance criteria with an = average microorganism R^2^ value of 0.9914. A total of nine microorganisms were tested. Seven microorganisms were tested once each and two microorganisms, *S. maltophilia* and *B. spizizenii* (heat-shocked), were tested twice.

**Table 24.** Linearity results—microorganisms

Microorganism	R^2^ result
*P. paraeruginosa*	0.9983
*B. spizizenii* (heat-shocked)	0.9994
	0.9995
*R. pickettii* (strain No. 1)	0.9896
*S. aureus*	0.9961
*S. maltophilia*	0.9979
	0.9962
*P. fluorescens* (starved)	0.9978
*E. coli*	0.9972
*S. paucimobilis*	0.9421
*B. cepacia*	0.9915
Average	0.9914

Each microorganism individually met the linearity acceptance criteria with R^2^ values of 0.9025 or higher with the lowest R^2^ value calculated to be 0.9915 (*B. cepacia*). *S. maltophilia* was tested on two separate days and R^2^ values closely matched with calculated values of 0.9979 and 0.9962. Additionally, the heat-shocked *B. spizizenii* inoculum was tested on two separate occasions and the R^2^ values from these analyses were also extremely close at 0.9994 and 0.9995.

Linear reggression analyses for *B. spizizenii* and *P. glucanolyticus* were also calculated for informational purposes. These data demonstrated acceptable linearity despite the active population decline as the R^2^ values were calculated to be 0.9975 and 0.9755, respectively*.* Therefore, although the cultures were actively dying, 7000RMS was still demonstrating linearity which further supports that these microorganisms simply could not be cultivated (i.e., VBNC). These data show that the 7000RMS could be useful in pharmaceutical water systems for system maintenance applications where a microorganism population is expected to decline after exposure to the sanitizing event.

Additionally, for informational purposes, a linear regression was performed from the mixed culture of *S. aureus/P. paraeruginosa* analysis*.* This mixed culture demonstrated excellent linearity with a calculated R^2^ value of 0.9974. This demonstrates that data from 7000RMS could be useful in pharmaceutical water systems for the detection of biofilms, as they are often populated by several different species of microorganisms.

The results of testing using beads are summarized in Table 25. Due to the syringe capacity, two different bead challenges, a low range and high range, were analyzed for linearity both individually and as a combined data set to ensure that the full operational range was adequately covered for online mode testing. The lowest individual R^2^ value was calculated to be 0.9077, derived from testing the lower concentrations using a 100 mL volume and analyzed using online mode. However, when data was combined with the higher concentration data set, the calculated R^2^ value was 0.9937. Testing using the 10 mL volume followed this same pattern where the lower concentration was slightly less linear than the higher concentration.

**Table 25.** Linearity for beads

Test procedure	Volume	Test	Bead concentration	R^2^ result	Combined R^2^
Sample mode	1 mL	Challenge No. 1	<1–10 105	0.9989	0.9804
		Challenge No. 2	<1–10 032	0.9992	
Online mode	10 mL	Syringe No. 1	5–1001	0.9930	0.9947
		Syringe No. 2	51–10 134	0.9986	
Online mode	100 mL	Syringe No. 1	25–1006	0.9077	0.9937
		Syringe No. 2	251–10 030	0.9927	

Sample mode testing was performed over the full range using two different inoculum preparations. All testing both individually and combined resulted in acceptable linearity.

### Operational Range

The range for the 7000RMS was found to be 25–10 030 counts per 100 mL. Table 26 summarizes the lowest and highest concentrations tested where accuracy, precision, and linearity acceptance criteria were met. Although the 7000RMS was accurate down to 15 beads per 100 mL, the lower bound range was defined by precision and linearity as these parameters were met at 25 beads per 100 mL.

**Table 26.** Operational (dynamic) range—bead results

Accuracy		Precision		Linearity	
Lowest concn met	Highest concn met	Lowest concn met	Highest concn met	Lowest concn met	Highest concn met
15	10 030	25	10 030	25	10 030

Although the interval between the lower and upper limit is sufficient for the purposes of detecting bioburden in PW water, testing did not fully challenge the upper range for the 7000RMS. It is likely much higher than the range validated and bounded by where coincidence error occurs.

### Performance Equivalence

Table 27 lists all validation parameters tested during this validation. However, performance equivalence requires that both the 7000RMS and the MF method test the same sample in the same manner. As such, no comparisons were made for several parameters including LOQ, linearity, and range.

**Table 27.** Selection of performance equivalence parameters

Parameter	7000RMS	MF	Performance equivalence possible?	Expectation for 7000RMS performance compared to MF
Accuracy	X	X	Yes	Equivalent performance not expected due to non-equivalence of AFU to CFU when strict %recovery applied.
Specificity	X	X	Yes	Equivalent or better performance
Precision	X	X	Yes	Equivalent or better performance
LOD	X	X	Yes	Equivalent or better performance
LOQ	X		No	
Linearity	X		No	
Range	X		No	

It is important to note that per USP <1223>, performance equivalence does not require that *all* validation parameters result in equivalent or better to performance to the current method (MF) in the cases where there are advantages in using the alternative method (7000RMS). These advantages have been summarized in Table 1 at the beginning of this article; the USP provides some leeway in allowing the microbiologist to determine which criteria are necessary to demonstrate performance equivalence.

This validation was not specifically designed to compare results of LOQ, range, and linearity between the MF and the 7000RMS. In the MF method, these parameters are constrained by the accuracy of the count on an agar plate. The accepted range for counting colonies accurately is 25–250 for bacteria and *Candida albicans* (7). Therefore, while no direct testing of these parameters was performed, the 7000RMS was shown to have a range of 25–10 030 and was, therefore, superior to the MF method.

Table 28 provides the results from each parameter and show that results meet acceptance criteria since the 7000RMS was determined to have equivalent or superior performance for three of the four parameters.

**Table 28.** Equivalency results

Parameter	Equivalent or better performance?
Specificity	Yes—equivalent
Precision	Yes—equivalent
LOD	Yes—superior
Accuracy	No

### Correlation

The analysis was conducted using both log 10 transformed and untransformed data, which concluded that the degree of correlation of 7000RMS AFU results to MF CFU counts were microorganism-dependent.

**Figure 6.**
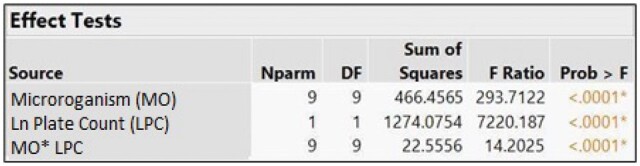
Outcome of ANOVA analysis.

The adjusted 7000RMS AFU result and MF method CFU result for microorganisms at the five different concentrations were combined and transformed to natural log (Ln). A one-way ANOVA was performed to determine if there was a statistically significant difference in the microorganism slope and y-intercept result. The results in Figure 6 indicate that the y-intercepts (Microorganism <0.0001) and slopes (Microorganism*Ln Plate Count CFU <0.0001) are statistically different for the different microorganisms (*see* asterisk * in Figure 6).

**Figure 7.**
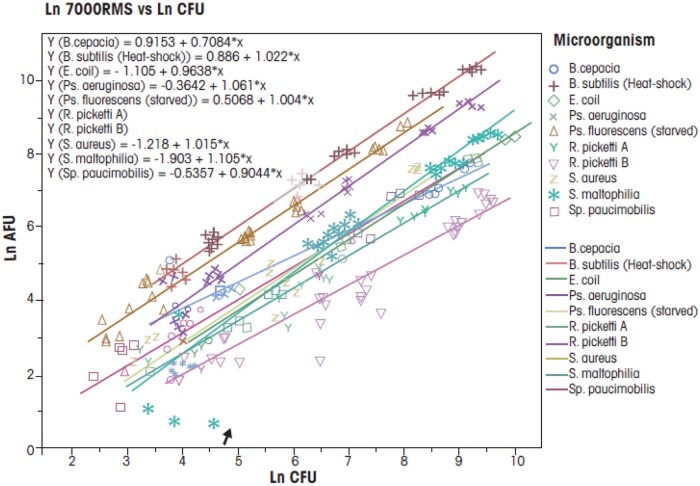
Transformed plot.

This difference means that one single correlation factor cannot be applied to all organisms as the data is dependent on the type of microorganism that caused each count. As such, the outcome of one method based on the results of the other method using the numerical result reported cannot be predicted. This point is further illustrated by examining the difference in all the linear regression lines in a single plot. The plot of transformed results in Figure 7 displays the degree of different y-intercepts and different slopes.

Table 29 shows that, while most microorganisms show some degree of correlation for both transformed and untransformed data, *Burkholderia cepacia* has a good correlation using untransformed data with an R^2^ value of 0.9881; however, there is a lack of fit when the data is transformed to log10 with an R^2^ value of 0.8564. Conversely, *Staphylococcus aureus* has a lack of fit using untransformed data with an R^2^ value of 0.8326 but shows a good correlation when the data are transformed to log10 with an R^2^ value of 0.9445.

**Table 29.** Correlation values (R^2^) transformed and non-transformed

Microorganism	Untransformed R^2^		Transformed R^2^	
	R^2^ value	≥0.9025	R^2^ value	≥0.9025
*B. cepacia*	0.8564	No	0.9881	Yes
*B. spizizenii* (heat-shocked)	0.9922	Yes	0.9846	Yes
*E. coli*	0.9795	Yes	0.9860	Yes
*P. paraeruginosa*	0.9787	Yes	0.9758	Yes
*Ps. fluorescens*	0.9660	Yes	0.9622	Yes
*R. pickettii* (strain No. 1)	0.9633	Yes	0.9684	Yes
*R. pickettii* (strain No. 2)	0.9054	Yes	0.9448	Yes
*S. aureus*	0.9445	Yes	0.8326	No
*S. maltophilia*	0.9446	Yes	0.9826	Yes
*Sp. paucimobilis*	0.9442	Yes	0.9137	Yes

The 7000RMS instrument provides bioburden counts in AFU but cannot provide the microorganism identification that resulted in each reported AFU. In conclusion, the total reported AFU within a given volume (or time frame) could arise from one or more unknown microorganisms on the 7000RMS system.

### Points to Consider for Future Testing

The execution of the 7000RMS validation presented here utilized techniques not common to the microbiology laboratory. The following points to consider been developed to aid other laboratories conducting testing using laser-induced bio-fluorescent particle-counting OWBA instruments.

When preparing for and executing the validation of the 7000RMS in a laboratory setting several challenges were noted and are worth sharing. Even though the 7000RMS is considered a commercial off-the-shelf (COTS) system, there may be significant prework needed to understand the operation of the 7000RMS in conjunction with the preparation and planning that is required to execute testing and data collection. Early adopters may consider working with the vendor on the design and implementation of the validation process.

The 7000RMS provides precise results but is influenced by testing conditions at lower concentrations. Therefore, it is important to prioritize cleaning of sample bottles and tubes before the testing. The proper cleaning procedure ensures adequate particulate control since extraneous particles can influence overall particle counts. Additionally, the cleaning and sanitization procedures should be closely adhered to in order to prevent cross-contamination. For multi-use materials like the syringe, particles or samples may be carried over to subsequent tests. To ensure the syringe is sanitized and cleaned, the syringe can be disassembled, sonicated with hydrogen peroxide, transferred into the Biological Safety Cabinet (BSC) with the UV lamp turned on, and left overnight. In multi-use bottles, it is recommended to rinse and wash with ultra-pure water for 30 s and to repeat this three times.

One may also consider using median rather than mean to calculate background since the mean is subjected to a skewed distribution in the case of extreme observations.

A typical laboratory is not optimized for innovative technology execution like the 7000RMS validation. During the execution of the validation, the instruments needed were all in separate laboratory spaces requiring excess movement of personnel and materials between various areas of the laboratory. On some test days, an additional person was needed to hand off the samples collected from the 7000RMS to the BSC laboratory for MF testing.

Daily testing execution typically required 6–8 h. More than half of this time was needed for setup, preparation, and teardown. Approximately 2–3 h were needed to prepare materials and the 7000RMS and another hour was needed for teardown (e.g., post-use sanitization of the 7000RMS, waste disposal).

The GenPure water system was fed from the laboratory deionized water loop. To reduce the potential for biofilm formation/contamination and to maintain sanitary conditions, the 7000RMS was left connected to the water and running throughout the validation period. Heavy water use in the laboratory may lead to interruptions on the 7000RMS when the water flow becomes too low. This could lead to delays in the acquisition of data. Furthermore, the 7000RMS must operate within a pressure range of 20–80 psig or 2–5.5 bar.

Laboratory waste streams were impacted requiring the engagement of the firm’s Environmental Health Safety and Sustainability group to ensure waste was appropriately accumulated and disposed of. This included developing a new waste stream for collecting peroxides used during 7000RMS sanitization and adding bins/collection times for the increase in recyclable waste (bottles), biohazardous waste (plates, cultures, etc.), and sharps (glass tubes).

Validation guidance documents USP <1223> (1), EP 5.1.6 (2), and PDA Technical Report 33 (3) do not account for AFU and CFU non-equivalence in many of the validation attribute examples as written. For this reason, a bead was used as a microorganism surrogate to challenge the 7000RMS detection capabilities. The use of a bead in a microbial validation is a novel approach requiring clear and sound scientific justification.

## Conclusions

The testing and results described herein are one of the first end user laboratory challenges that demonstrate that the 7000RMS can meet the validation criteria for a quantitative method. It is recognized that the path to validating an OWBA instrument can vary based on the end user's intended use and different water systems. Although the validation approach provided in this publication was specific to one end user with a particular set of requirements, this approach may serve as guidance to other end users when conducting similar testing.

The validation study was completed in a laboratory setting and did not include any examination of the 7000RMS instrument within the manufacturing water systems. For full implementation, additional steps are still required, including performance qualification, to demonstrate the suitability of the instrument in the manufacturing area and determining meaningful control values that will signal departures from a defined baseline.

Even though this was a first user-based study, this validation can be easily replicated as Mettler-Toledo provides the injection system as a package. Continuous online monitoring can offer the end user assurance of required water quality while also detecting system excursions in a timely manner. Real-time monitoring of inert and biological particles (AFU) can reduce risk by enhancing water system monitoring with improved detection and enumeration of VBNCs over the MF method for some microorganisms (*P. glucanolyticus* and *B. spizizenii*) and improving the overall assurance of specifications within a pharmaceutical water system.
